# A rare presentation of gastric phytobezoar: Simultaneous bleeding and perforation. combined laparoscopic and endoscopic approach. Report of a case

**DOI:** 10.1016/j.ijscr.2023.108841

**Published:** 2023-09-18

**Authors:** Giuseppe Di Buono, Gaia Russo, Giuseppe Amato, Matilde Micheli, Girolamo Geraci, Antonino Agrusa

**Affiliations:** Department of Surgical, Oncological and Oral Sciences, University of Palermo, Palermo, Italy

**Keywords:** Peritonitis, Gastric perforation, Laparoscopy, Gastric phytobezoar, Emergency surgery

## Abstract

**Introduction:**

Bezoars are intraluminal conglomerates of indigestible foreign materials that accumulate in the gastrointestinal tract. We describe our experience with a patient with gastric perforation and concomitant gastric haemorrhage with severe anaemia, in whom we successfully extracted a giant gastric phytobezoar by cooperative laparoscopic and endoscopic surgery.

**Case presentation:**

A 68-year-old man was admitted with melena and septic shock. CT scan revealed a gastric perforation. We performed a combined laparoscopic and endoscopic approach with gastrotomy, removal of the phytobezoar and laparoscopic gastric suture. The suture was examined for leakage with the endoscopic hydropneumatic test to obtain direct vision of the suture and no evidence of leakage by insufflation of the area.

**Discussion:**

Gastric bezoars can be managed conservatively, endoscopically or surgically. Endoscopic removal, if effective, would be an attractive alternative for bezoar treatment. Usually endoscopic attempts are unsuccessful because of the large size of the bezoar and the difficulty in fragmentation. The laparoscopic approach for bezoar seems to have better postoperative outcomes. The main criticisms of the technique are abdominal spillage with risk of contamination as well as longer operative times.

**Conclusion:**

In our case we simultaneously performed laparoscopic surgery and endoscopic operative procedure in accordance with the principles of laparoscopic and endoscopic cooperative surgery to treat the gastric bezoar in order to overcome the limits of a single technique.

## Highlights

• Bezoars are intraluminal conglomerates of indigestible foreign materials that accumulate in the gastrointestinal tract.
• We present a case of gastric perforation and bleeding due to a large phytobezoar.
• The combined laparoscopic and endoscopic approach overcomes the limits of a single technique.

## 1 Introduction

Bezoars are intraluminal conglomerates of indigestible foreign materials that accumulate in the gastrointestinal (GI) tract. A variety of materials received orally, intentionally or accidentally, can form these indigestible masses. Consequently, several types of bezoars have been described, including phytobezoars (fibres, fruit remains, vegetable peels, etc.), trichobezoars (ingested hair), pharmacobezoars (drugs), lactobezoars (milk proteins in infants and milk-fed infants) and others [1,2]. Bezoars can form in any segment of the GI tract. However, the stomach is the most common organ of bezoar formation [3]. Most intragastric bezoars occur in adolescents and young women with psychiatric disorders. In adults, on the other hand, most intragastric bezoars are related to gastroparesis, anatomical abnormalities and previous gastric surgery that reduces gastric motility [4]. Many approaches have been proposed for the treatment of bezoars, such as medical therapy with agents and enzymes that promote gastric motility and/or gastroscopic fragmentation [5]. But when these treatments fail, surgical removal is necessary. Traditionally the surgical treatment of bezoars has been done by laparotomy approach, but due to the results published in recent studies, the laparoscopic approach is more accepted as an option every day of surgical treatment. With regard to the removal of the gastric bezoar, although only a few cases of minimally invasive technique are reported in the literature, laparoscopic surgery has been performed in the past and the laparoscopic approach has been shown to be safe and feasible [6,7]. The laparoscopic method presents a risk of intra-abdominal contamination when the gastric bezoar is retrieved from the gastric lumen into the peritoneal cavity. We describe our experience with a patient with gastric perforation and concomitant gastric haemorrhage with severe anaemia, in whom we successfully extracted a giant gastric phytobezoar by cooperative laparoscopic and endoscopic surgery [8]. This case was reported in line with the SCARE criteria and PROCESS guidelines [9,10].

## 2 Case presentation

**Fig. 1:**
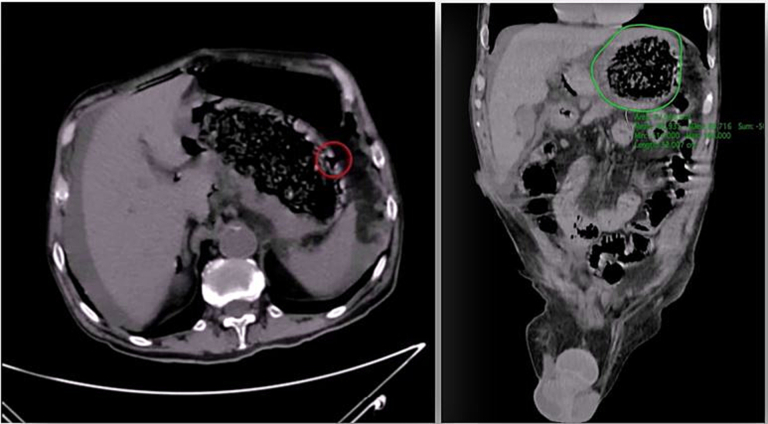
CT abdominal scan with free fluid and air in the abdominal cavity. The stomach appeared full of dense material with thickened walls.

**Fig. 2:**
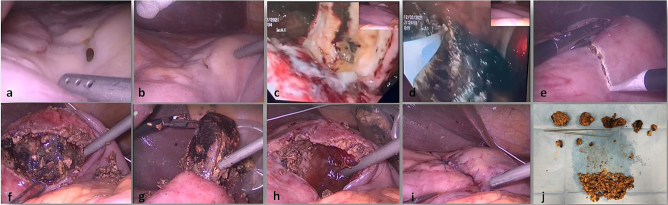
Intraoperative findings (explanation in the text).

**Fig. 3:**
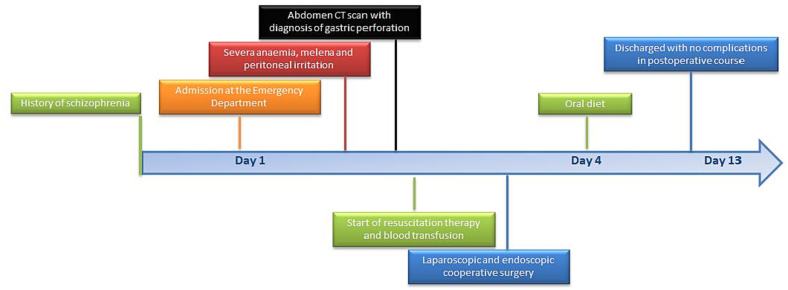
Timeline of diagnostic assessment, procedures and outcomes.

A 68-year-old man was admitted to the our emergency department with acute abdomen and septic shock with hypotension, tachycardia, tachypnoea, loss of consciousness (blood pressure 87/34 mm Hg; heart rate 150 bpm in sinus rhythm; respiratory rate 21 breaths per minute; Glasgow Coma Scale 6) and severe anaemia (Hb 5.5 g/dL). On physical examination the skin and mucous membranes were pale and the abdomen was distended without sound, with diffuse tenderness and signs of peritoneal irritation. Rectal examination revealed melena. Other laboratory tests on admission were as follows: haematocrit 17.7 %; leucocyte count 14,390/μL (neutrophils 90 %); platelets 322,000/μL; C-reactive protein 59.41 mg/L; creatinine 1.25 mg/dL; sodium 140 mmol/L; potassium 3.39 mmol/L. The medical history, with the exception of schizophrenia, was not relevant for the current disorder. Computed tomography (CT) of the abdomen showed the presence of diffuse thickening of the stomach wall, free air in the abdomen and a moderate amount of free fluid. These signs were consistent with pneumoperitoneum caused by the gastric perforation (Fig. 1). Support began with conservative therapy: oxygen therapy and resuscitation with fluids (crystalloids) and vasopressors (noradrenaline). The patient received antibiotic therapy (ceftriaxone 2 g and metronidazole 500 mg) and a transfusion of 2 packed red blood cells (pRBCs). After haemodynamic stabilisation and evaluation by the multidisciplinary team, it was decided to perform laparoscopic surgery [11]. Under general anaesthesia, the patient was placed in the classic French position and the surgeon stood between the patient's legs. We induced the pneumoperitoneum with an open transumbilical laparoscopy. Three more ports were inserted under vision in the right flank (5 mm), left hypochondrium (10 mm) and epigastric region (5 mm). On laparoscopic exploration, a massive peritonitis was found with free fluid in the supra- and sub-mesocolic compartments, which was taken for microbiological examination. A 2 cm perforation in the anterior wall of the prepyloric region of the stomach was identified (Fig. 2a). Despite the anaemia, perforation and thickening of the stomach wall (Fig. 2b), there did not appear to be a gastric tumor. In order to have a more certain diagnosis, it was decided to perform an intraoperative gastroscopy and a large phytobezoar (10 × 5 cm) was found occupying the entire lumen of the stomach. Four large gastric ulcers were also found, with signs of recent bleeding (Fig. 2c–d). Due to the large size and hardness of the phytobezoar, its fragmentation and endoscopic removal were unsuccessful and surgical removal was therefore performed. A 5 cm linear gastrotomy was performed in the anterior wall of the stomach with a monopolar hook (Fig. 2e) and the phytobezoar was immediately visible. It was fragmented with difficulty and placed in a laparoscopic pouch (Fig. 2f–g–h–j). The gastrotomy was closed in a double layer with a 3-0 V-lock suture (Fig. 2i). The suture was examined for leakage with the endoscopic hydropneumatic test to obtain direct vision of the suture and no evidence of leakage by insufflation of the area. We left a nasogastric tube and an abdominal drainage in the subhepatic region. The duration of the surgery was 180 min. At the end of the surgical procedure the patient was transferred to the intensive care unit (ICU). He maintained the nasogastric tube for 3 days after the operation, then started the oral diet. He was discharged on postoperative day 12 after a psychiatric consultation. The timeline of the diagnostic evaluation, surgical procedure and outcome is described in Fig. 3. The patient and his legal guardians gave their informed consent for the publication of this clinical case.

## 3 Discussion

Bezoars are masses of undigested food particles and fibrous materials present in the gastrointestinal system. They can be found anywhere in the gastrointestinal tract, although they occur most frequently in the stomach and small intestine [12,13]. Among bezoars, phytobezoars are the most common and best known group. Elderly patients and those with diabetes mellitus or a history of gastrointestinal surgery are more likely to develop bezoars due to altered gastric motility. Psychiatric conditions are also risk factors for the development of bezoars [14,15]. Bezoars can cause abdominal discomfort, fullness or pain, difficulty swallowing or anorexia. Furthermore, symptoms related to GI bleeding, such as anaemia, melena or recthorragia and hematemesis can result from ulcers and mucosa necrosis from increased bezoar-related intraluminal pressure [16]. In the rare cases of complete intestinal obstruction or perforation, the patient present serious manifestations of acute abdomen, vomiting, abdominal distension, hypotension, mental disorientation and shock [17,18]. Gastric bezoars can be managed conservatively, endoscopically or surgically. Endoscopic removal, if effective, would be an attractive alternative for bezoar treatment. However, usually endoscopic attempts are unsuccessful because of the large size of the bezoar and the difficulty in fragmentation [19]. Furthermore, endoscopic fragmentation can cause the migration of the fragmented part and repeated endoscopic maneuvers might be needed to remove the remnant segments responsible of pressure ulceration, esophagitis and esophageal perforation [20]. Surgery is required to remove the very large gastric bezoars and those who present with intestinal obstruction. Laparotomy for bezoar removal is the most commonly described technique in literature. Minimally invasive techniques are possible and only a few cases have been reported on laparoscopic management of gastric bezoar. The first successful laparoscopic removal of a gastric bezoar was reported in 1998 by Nirasawa et al. [7] in a pediatric patient. The authors performed an 8-cm laparoscopic gastric incision placing the bezoar in a plastic bag and then removing it through a suprapubic minilaparotomy. Since then, some successful laparoscopic cases have been reported, primarily in adults and adolescents [[21], [22], [23]]. In a retrospective study conducted by Yau et al. [24] the laparoscopic approach for bezoar induced small bowel obstruction was shown to have better postoperative outcomes. The patients treated laparoscopically had fewer complications, shorter hospital stays and faster return of bowel function. Though laparoscopy presents many advantages, the main criticism of the technique are abdominal spillage with risk of contamination as well as longer operative times. The minimization of spillage can be accomplished by careful transfer of the bezoar and associated debris to a sturdy endobag. Once the specimen is removed, careful inspection of the abdomen with copious irrigation prevents potential intraabdominal complications. To overcome the disadvantages of conventional laparoscopic treatments, in our case we simultaneously performed laparoscopic surgery and endoscopic operative procedure in accordance with the principles of laparoscopic and endoscopic cooperative surgery (LECS) to treat the gastric bezoar. Laparoscopic and endoscopic cooperative surgery (LECS) is a newly developed concept for tumor dissection of the gastrointestinal tract that was first investigated for local resection of gastric gastrointestinal stromal tumors (GIST) [25,26] and also for combined resection of complex right colon polyps [27]. This approach for a lesion is as follows: the lesion is recognized directly under endoscopic visualization and fully resected in an en bloc full-thickness manner endoscopically and/or laparoscopically. The defect of the stomach wall is sutured through laparoscopic linear staplers or laparoscopic hand-suturing techniques [28]. In literature we found only one study documenting the removal of gastric bezoars by laparoscopic and endoscopic surgery in accordance with the principles of laparoscopic and endoscopic cooperative surgery [29]. In our case, the use of endoscopy allowed us to see the true dimension of the bezoar and thanks to the use of insufflation we were able to test the gastric suture (intraoperative air leak test).

## 4 Conclusion

Laparoscopic and endoscopic cooperative surgery can be considered a safe technique for the removal of gastric bezoars that combines the advantages of the endoscopic and laparoscopic technique and in the future it could become an effective alternative treatment.

## Consent to publish

Written informed consent was obtained from the patient for publication of this case report and accompanying images. A copy of the written consent is available for review by the Editor-in-Chief of this journal on request.

## Provenance and peer review

Not commissioned, externally peer-reviewed.

## Ethical approval

No ethical approval needed for this manuscript.

## Funding

This article did not receive sponsorship for publication.

## Guarantor

Di Buono Giuseppe
Agrusa Antonino.

## Research registration number

Not applicable.

## CRediT authorship contribution statement

Di Buono Giuseppe: study design, data collections, data analysis and writing
Russo Gaia: study design, data collections, data analysis and writing
Amato Giuseppe: study design, data collections, data analysis and writing
Micheli Matilde: study design, data collections, data analysis and writing
Geraci Girolamo: study design, data collections, data analysis and writing
Agrusa Antonino: study design, data collections, data analysis and writing.

## Declaration of competing interest

The authors declare that they have no competing interests.

## References

[1] Eng K., Kay M. (2012). Gastrointestinal bezoars: history and current treatment paradigms. Gastroenterol. Hepatol. (N. Y.).

[2] Krausz M.M., Morial E.Z., Ayalon A. (1985). Surgical aspects of gastrointestinal persimmon phytobezoar treatment. Am. J. Surg..

[3] Koulas S.G., Zikos N., Charalampous C., Christodoulou K., Sakkas L., Katsamakis N. (2008). Management of gastrointestinal bezoars: an analysis of 23 cases. Int. Surg..

[4] Chacko A., Masters B.I., Isles A. (2017). Giant gastric bezoar complicating congenital esophageal atresia repaired by gastric interposition—a case report. Front. Pediatr..

[5] Benes J., Chmel J., Jodl J. (1991). Treatment of a gastric bezoar by extracorporeal shock wave lithotripsy. Endoscopy..

[6] Fraser J.D., Leys C.M., St Peter S.D. (2009). Laparoscopic removal of a gastric trichobezoar in a pediatric patient. J. Laparoendosc. Adv. Surg. Tech. A.

[7] Nirasawa Y., Mori T., Ito Y., Tanaka H., Seki N., Atomi Y. (1998). Laparoscopic removal of a large gastric trichobezoar. J. Pediatr. Surg..

[8] Ntourakis D., Mavrogenis G. (Nov 21 2015). Cooperative laparoscopic endoscopic and hybrid laparoscopic surgery for upper gastrointestinal tumors: Current status. World J. Gastroenterol..

[9] Agha R.A., Franchi T., Sohrab C., Mathew G., Kirwan A., Thomas A. (2020). The SCARE 2020 guideline: updating consensus Surgical Case Report (SCARE) guidelines. Int. J. Surg..

[10] Agha R.A., Borrelli M.R., Farwana R., Koshy K., Fowler A., Orgill D.P., SCARE Group (2018). The PROCESS 2018 statement: updating consensus Preferred Reporting Of CasE Series in Surgery (PROCESS) guidelines. Int. J. Surg..

[11] Agrusa A., Romano G., Dominguez L.J., Amato G., Citarrella R., Vernuccio L., Di Buono G., Sorce V., Gulotta L., Galia M., Mansueto P., Gulotta G. (2016). Adrenal cavernous hemangioma: which correct decision making process?. Acta Med. Mediterranea..

[12] Wadlington W.B., Rose M., Holcomb G.W. (1992). Complication of trichobezoars: a 30 year experience. South. Med. J..

[13] Byrme W.J. (1994). Foreign bodies, bezoars and caustic ingestion. Gastrointest. Endosc. Clin. North Am..

[14] Cifuentes Tebar J., Robles Campos R., Parrilla Paricio P. (1992). Gastric surgery and bezoars. Dig. Dis. Sci..

[15] Sanders M.K. (2004). Bezoars: from mystical charms to medical and nutritional management. Pract. Gastroenterol..

[16] Iwamuro M., Tanaka S., Shiode J. (2014). Clinical characteristics and treatment outcomes of nineteen Japanese patients with gastrointestinal bezoars. Intern. Med..

[17] Altintoprak F., Dikicier E., Deveci U. (2012). Intestinal obstruction due to bezoars: a retrospective clinical study. Eur. J. Trauma Emerg. Surg..

[18] Pujar K.A., Pai A.S., Hiremath V.B. (2013). Phytobezoar: a rare cause of small bowel obstruction. J. Clin. Diagn. Res..

[19] Wang Y.G., Seitz U., Li Z.L., Soehendra N., Qiao X.A. (1998). Endoscopic management of huge bezoars. Endoscopy.

[20] Dumonceaux A., Michaud L., Bonnevalle M., Debeugny P., Gottrand F., Turck D. (1998). Trichobezoars in children and adolescents. Arch. Pediatr..

[21] Song K.Y., Choi B.J., Kim S.N., Park C.H. (2007). Laparoscopic removal of gastric bezoar. Surg. Laparosc. Endosc. Percutan. Tech..

[22] Yao C.C., Wong H.H., Chen C.C., Wang C.C., Yang C.C., Lin C.S. (2000). Laparoscopic removal of large gastric phytobezoars. Surg. Laparosc. Endosc. Percutan. Tech..

[23] Kanetaka K., Azuma T., Ito S., Matsuo S., Yamaguchi S., Shirono K., Kanematsu T. (2003). Two-channel method for retrieval of gastric trichobezoar: report of a case. J. Pediatr. Surg..

[24] Yau K.K., Siu W.T., Law B.K., Cheung H.Y., Ha J.P., Li M.K. (2005). Laparoscopic approach compared with conventional open approach for bezoar-induced small-bowel obstruction. Arch. Surg..

[25] Hiki N., Nunobe S., Matsuda T., Hirasawa T., Yamamoto Y., Yamaguchi T. (2015 Jan). Laparoscopic endoscopic cooperative surgery. Dig. Endosc..

[26] Di Buono G., Maienza E., Buscemi S., Bonventre G., Romano G., Agrusa A. (2020). Combined endo-laparoscopic treatment of large gastrointestinal stromal tumor of the stomach: report of a case and literature review. Int. J. Surg. Case Rep..

[27] Yan J., Trencheva K., Lee S.W., Sonoda T., Parul Shukla, Milsom J.W. (June 2011). Treatment for right colon polyps not removable using standard colonoscopy: combined laparoscopic-colonoscopic approach. Dis. Colon Rectum.

[28] Wang H., Cao L., Zheng K., Zhao Y. (2018 Dec). Laparoscopic endoscopic cooperative surgery for gastrointestinal stromal tumors. Surg. Laparosc. Endosc. Percutan. Tech..

[29] Kurosu T., Tanabe S., Hasegawa R., Yano T., Wada T., Ishido K., Azuma M., Katada C., Koizumi W., Moriya H., Yamashita K. (2018 Dec). A giant trichobezoar extracted by laparoscopic and endoscopic cooperative surgery (LECS). Endosc. Int. Open.

